# Recent Developments towards Commercialization of Metal Matrix Composites

**DOI:** 10.3390/ma13122828

**Published:** 2020-06-24

**Authors:** Dae-Young Kim, Hyun-Joo Choi

**Affiliations:** School of Advanced Materials Engineering, Kookmin University, Seoul 02707, Korea; kdy6603@kookmin.ac.kr

**Keywords:** metal matrix composites, aluminum nitride, nitridation, exothermic reaction, NISFAC process, Diamond/Cu composites, neutral salt spray, corrosion behavior, thermal conductivity, SiCp/Al composites, scratch test, 3D finite element model, material removal, surface defect

## Abstract

Metal matrix composites (MMCs) are promising alternatives to metallic alloys. Their high strength-to-weight ratios; high temperature stabilities; and unique thermal, electrical, and chemical properties make them suitable for automotive, aerospace, defense, electrical, electronic, energy, biomedical, and other applications. The wide range of potential combinations of materials allows the properties of MMCs to be tailored by manipulating the morphology, size, orientation, and fraction of reinforcement, offering further opportunities for a variety of applications in daily life. This Special Issue, “Metal Matrix Composites”, addresses advances in the material science, processing, material modeling and characterization, performance, and testing of metal matrix composites.

Metal matrix composites (MMCs) consist of a metallic matrix and a foreign reinforcement, such as ceramic, metallic, or carbon materials. Therefore, their properties are responsive to the type, shape, size, volume, or dispersion of the reinforcement. MMCs may overcome the limitations of conventional alloys, such as unstable microstructures at high temperatures and trade-offs between features like conductivity and strength. Given these advantages, the global market for MMCs is expected to reach USD 433.3 million by 2022 [1]. An increasing need for high-performance composite metals is expected from the automotive, aerospace, and marine industries because of the importance of weight reduction to addressing fuel-efficiency and environmental issues.

Nevertheless, high manufacturing costs and limited technical expertise are expected to pose a challenge for MMC market growth. Increasing the market share of the MMCs competing with light metal alloys and plastic composites requires both greater mechanical performance and cost-effectiveness. Conventional MMC manufacturing processes can be categorized as liquid-state or solid-state techniques. Liquid-state techniques, which include stir casting and infiltration, proceed at temperatures above the melting point of the matrix material. Solid-state techniques are conducted below the melting point of the matrix material, with powder metallurgy (PM) being the representative technique. Stir casting [2,3] is a well-known, economical route for processing MMCs, but it is limited to the dispersion of small-sized reinforcement in the matrix and the development of composites containing continuous fibers. Infiltration [4,5] enables near-net-shape forming of composites with high volumes (up to ~70 vol.%) of reinforcement, but the process involves a costly preform synthesis prior to infiltration. Furthermore, these liquid-state techniques limit the type, shape, or size of the reinforcement because of the formation of unfavorable reactants like oxides or carbides at high processing temperatures. The PM route provides great flexibility in the selection of the type, shape, size, and volume of the reinforcement, as well as superb mechanical and thermal properties stemming from the uniform dispersion of the reinforcement with a clean interface. However, the high cost of the PM process limits its industrial use.

Recently, nanocomposite materials, which contain nano-scale reinforcement such as graphene, carbon nanotubes, and h-BN, have been actively studied. However, papers on technologies to overcome bottle-neck for expanding the industrial application of conventional composites are relatively less attended. This Special Issue introduces various efforts to increase the price competitiveness, reliability and applicability of conventional MMC. This Special Issue consists of four articles covering topics ranging from a new cost-effective manufacturing process for MMCs to a numerical and experimental effort for enhancing reliability of MMCs. A series article in this Special Issue reports a new, cost-effective MMC manufacturing process that overcomes the limitations of the existing processes [6,7]. In the new process, aluminum powder and ceramic reinforcement mixtures are heated in nitrogen gas, thus allowing the exothermic nitridation reaction to partially melt the aluminum powder, which assists the composite densification and improves the wetting between the aluminum and the ceramic. Achieving the degree of nitridation needed to form sufficient molten aluminum is key to producing sound, pore-free aluminum matrix composites (AMCs). This series paper describes the systematic investigation of the effects of the starting materials, including the chemical composition of the aluminum powder and the type, size, and volume fraction of the ceramic reinforcement, and the processing parameters, including the temperature, time, and nitrogen gas flow rate, on the nitridation behavior of aluminum powder.

On the other hand, this Special Issue also introduces efforts to enhance the reliability of conventional MMCs. One paper of this Special Issue introduces diamond-particle-reinforced copper matrix composites (Diamond/Cu) with promising applications in electronic packaging [8]. The reliability of the Diamond/Cu composites under extreme environmental conditions was evaluated by testing their corrosion behavior in a 5 wt.% NaCl neutral salt spray. Interestingly, the copper matrix was found to corrode mainly because of micro-galvanic corrosion, while the diamond and interface products did not corrode. Finally, Zhao et al. analyzed the sensitivity of the material removal process and the surface defect formation mechanism to the scratch depth during single-grit scratch tests of 50 vol.% SiCp/Al composites. The authors also developed a three-dimensional (3D) finite element model with more realistic 3D microstructures, particle–matrix interfacial behaviors, particle–particle contact behaviors, and particle-matrix contact behaviors that incorporates a Johnson–Holmquist–Beissel (JHB) model of SiC particles. Using the simulated and experimental results, the authors demonstrated the importance of the scratch depth to SiC particle removal and surface quality. We believe this Special Issue will act as a stepping-stone for commercialization in MMCs in various fields.
